# Ice-Templated Zwitterionic Sponge Hydrogels for Stable and Efficient Solar Desalination in High-Salinity Brines

**DOI:** 10.1021/acsami.6c00207

**Published:** 2026-02-02

**Authors:** Chang Zhang, Tiantian Yao, Jincui Gu, Peng Xiao, Tao Chen, Xuanzhou Chen, Louis D. Zhang, Yanhui Zhang

**Affiliations:** † School of Biological and Chemical Engineering, 199200 NingboTech University, Ningbo 315100, China; ‡ Fuan Pharmaceutical Group Ningbo Team Pharmaceutical Co., Ltd., Ningbo 315100, China; § Key Laboratory of Marine Materials and Related Technologies, Zhejiang Key Laboratory of Marine Materials and Protective Technologies, 74748 Ningbo Institute of Materials Technology and Engineering, Chinese Academy of Sciences, Ningbo 315201, China; ∥ School of Electrical and Computer Engineering, 1372 Georgia Institute of Technology, Atlanta, Georgia 30332, United States; ⊥ College of Engineering, 1076 University of Akron, Akron, Ohio 44325, United States; # Hangzhou Zheren Tech Co., Ltd., Hangzhou 311100, China

**Keywords:** ice-templated polymerization, zwitterionic sponge hydrogel, solar-driven steam generation, salt-tolerant desalination, antipolyelectrolyte effect

## Abstract

Solar-driven steam generation (SSG) offers a sustainable pathway for desalination, yet achieving temperature-regulated control over macroporous structures in salt-tolerant hydrogels remains a critical challenge. Here, we report a carbon black-coated PDMAPS sponge hydrogel (PDMAPS-CB-SH) fabricated via an ice-templated polymerization strategy, where the pore size and connectivity are tuned by regulating ice-crystal growth at different prefreezing temperatures. The optimized PDMAPS-CB-SH integrates abundant interconnected pores with the intrinsic antipolyelectrolyte effect of zwitterionic networks, enabling rapid water transport and stable swelling in brines up to 10 wt % NaCl. Upon incorporation of carbon black nanoparticles, the hydrogel evaporator achieves a high evaporation rate of 1.93 kg m^–2^ h^–1^ with an efficiency of 95.1% in seawater under 1 sun irradiation (1.0 kW m^–2^), and maintains stable evaporation performance under the tested high-salinity condition. Outdoor field tests further confirm its scalability, delivering 12.35 kg m^–2^ day^–1^ of freshwater with condensate quality meeting WHO drinking-water standards. This work establishes ice-templated zwitterionic sponge hydrogels as a versatile and scalable platform for efficient solar desalination, particularly under challenging high-salinity conditions.

## Graphical abstract

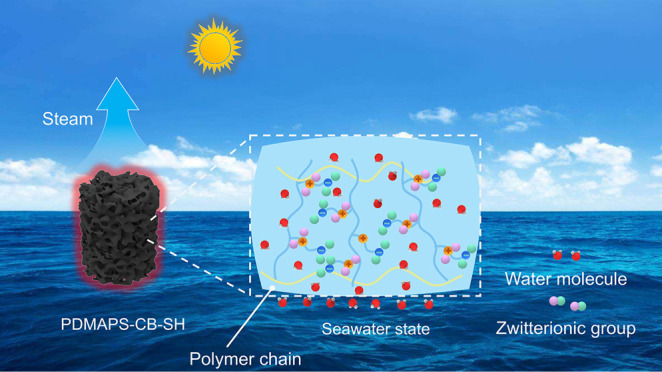

## 1 Introduction

Solar-driven steam generation (SSG) has emerged as an efficient and sustainable approach for freshwater production, relying on photothermal materials to harvest solar energy and drive localized water evaporation at the air–water interface.^1–8^ The condensed vapor provides potable water, making SSG a promising solution for regions suffering from water scarcity or emergency supply shortages. Central to an SSG system is the photothermal evaporator, which must simultaneously ensure: (i) high photothermal conversion efficiency, (ii) minimized thermal losses, and (iii) sufficient water transport to avoid salt accumulation and performance decay.

To enhance solar energy utilization and evaporation rates, researchers have designed evaporators using porous structures such as fabrics,^9–12^ biomass,^13–15^ aerogels^16–19^ and hydrogels.^20–23^ Among these, hydrogels are particularly attractive due to their soft, water-rich network and tunable composition. They can not only reduce the enthalpy change of water evaporation^24,25^ but also be engineered with functionalities such as salt resistance, antibacterial ability, and antifouling properties.^26–28^ However, conventional hydrogels suffer reduced swelling and water retention in saline environments because of the high osmotic pressure, limiting their practical use in seawater desalination. In contrast, zwitterionic hydrogels exhibit antipolyelectrolyte effects, enabling water absorption even in high-salinity brines, thus showing promise for robust evaporator design.^29–31^ In parallel with these structural strategies, advances in photothermal media (e.g., carbon black (CB)) have amplified interfacial heating due to its broadband absorption, thermal/chemical stability, and low cost. CB has consequently been widely adopted in SSG to boost light-to-heat conversion and sustain long-term operation.^32–34^

In addition to monomer selection, the internal structure of hydrogels critically influences water transport. Adequate water supply is essential to maintain continuous evaporation, as insufficient hydration reduces both volume and photothermal performance.^35,36^ Higher porosity facilitates water migration by providing interconnected pathways, thereby sustaining stable SSG. Existing strategies to fabricate porous hydrogels include: (i) template-assisted self-assembly,^37^ which creates well-defined pores but risks structural degradation during template removal; (ii) solvent-induced phase separation,^38,39^ which yields interconnected pores but may introduce secondary contamination; and (iii) freeze-drying,^40^ widely used for PVA-based hydrogels, though it offers limited control over pore size and involves complex procedures. Thus, developing hydrogel evaporators with controllable pore architecture and high stability remains an urgent challenge.

Here, we report the design of spongy polyzwitterionic hydrogels with tunable pore sizes prepared via ice-templated polymerization. Using the zwitterionic monomer [2-(methacryloyloxy)­ethyl]­dimethyl-(3-sulfopropyl) ammonium hydroxide (DMAPS), ice crystals serve as templates during polymerization to generate interconnected pores, whose size and morphology are regulated by the prefreezing temperature. We systematically investigated the effects of freezing conditions and polymerization time on pore structure, swelling behavior, and water transport. The optimized spongy PDMAPS hydrogels showed enhanced swelling kinetics, mechanical stability, and water supply capacity in both freshwater and brines. Incorporating CB as a photothermal agent further enabled stable evaporation performance under high-salinity conditions. The resulting PDMAPS-CB-SH hydrogel evaporators thus provide a promising platform for efficient solar desalination and sustainable freshwater production in challenging saline environments.

## 2 Experimental Section

### 2.1 Materials

[2-(Methacryloyloxy)­ethyl] dimethyl-(3-sulfopropyl) ammonium hydroxide (DMAPS) (purity ≥ 97%), ammonium persulfate (APS) (purity ≥ 98%), *N*,*N*,*N*′,*N*′-tetramethylethylenediamine (TEMED) (purity ≥ 96%), and *N*,*N*′-methylenebis acrylamide (BIS) (purity ≥ 96%) were purchased from Aladdin Shanghai Reagent Co. Ltd. Photothermal carbon black (CB) (30–45 nm, purity ≥ 97%) was obtained from Shanghai Macklin Biochemical Co., Ltd.

### 2.2 Synthesis of PDMAPS-CB Hydrogels

DMAPS (1.0 g), BIS (30 mg, cross-linker), and APS (10 mg) were dissolved in 5 mL of deionized (DI) water, followed by shaking and ultrasonication until a clear and transparent solution was obtained. Subsequently, TEMED (polymerization accelerator, 10 μL) and CB (0.1 g) were added, and the precursor solution was rapidly transferred into a cylindrical mold (3.5 cm in diameter, 5 cm in height). Polymerization was carried out at room temperature for 4 h. The resulting PDMAPS-CB hydrogel (PDMAPS-CB-HG) was removed from the mold and washed three times with DI water to eliminate residual monomers.

### 2.3 Preparation of PDMAPS-CB-SH

Similarly, DMAPS (1.0 g) monomers, BIS (30 mg), and APS (10 mg) were dissolved in 5 mL of DI water. The mixture was shaken and ultrasonicated until a clear, transparent solution was obtained. Subsequently, TEMED (polymerization accelerator, 10 μL) and CB (0.1 g) were added, and the precursor solution was rapidly poured into a cylindrical mold (3.5 cm in diameter, 5 cm in height). The mold was prefrozen at different temperatures (−40, −30, −20, −10, and 0 °C) for 0.5 h, followed by transfer to a −5 °C refrigerator for freezing and polymerization over 12 h. The resulting PDMAPS-CB-SH hydrogel sponge was removed from the mold and washed three times with deionized water to eliminate unreacted monomers.

### 2.4 Swelling Properties

The PDMAPS-CB-SH was immersed in NaCl solutions of varying concentrations (0, 3.5, 5, 15, 20, and 25 wt %) and allowed to reach equilibrium for 24 h. The swollen dimensions were measured using a vernier caliper, and the swollen mass was determined with an electronic balance.

### 2.5 Solar-Driven Evaporation

The swollen PDMAPS-CB-SH was cut into a cylindrical specimen with a diameter of ca. 35.0 mm and a height of ca. 10 mm. A polystyrene (PS) foam frame (inner diameter of ca. 35.0 mm, outer diameter of ca. 65.0 mm) was used to support the hydrogel on the surface of pure water or brine solutions with different concentrations, while also serving as thermal insulation. Solar-driven evaporation experiments were conducted under laboratory conditions at an ambient temperature of 25–30 °C and a relative humidity of 40–60%. The mass change of water was monitored using an electronic analytical balance with a resolution of 0.001 g.

### 2.6 Condensate Water Collection

PDMAPS-CB-HG and PDMAPS-CB-SH were placed on PS foam supports to enable stable flotation on the water surface. Outdoor experiments were carried out in Ningbo under clear weather conditions on June 4, 2025, from 9:00 to 18:00. A conventional transparent roof structure was employed as the condensation device. The entire evaporation–condensation setup was directly exposed to natural sunlight for 9 h to collect condensate water.

### 2.7 Characterizations

The microstructures of PDMAPS-CB-HG and PDMAPS-CB-SH were observed using a field-emission scanning electron microscope (FE-SEM, Hitachi S4800, 8 kV). Rheological measurements were performed on a Haake MARS III rheometer equipped with a 25 mm parallel plate, operated at frequencies from 0.1 to 100 Hz at 25 °C. The infrared spectra of PDMAPS-CB-SH were obtained using an attenuated total reflection Fourier transformed infrared spectrometer (ATR-FTIR). Ultraviolet–visible-near-infrared (UV–vis-NIR) transmittance and reflection spectra of PDMAPS-CB-HG and PDMAPS-CB-SH were measured with a spectrophotometer (PerkinElmer, Lambda 950, USA). The melting and evaporation behaviors were analyzed using a differential scanning calorimeter (DSC 214, Netzsch) under a nitrogen atmosphere with a flow rate of 40 mL min^–1^. The heating rates were set at 5 and 2 K min^–1^ for melting and evaporation, respectively. An infrared (IR) camera (FLIR E8) was used to record real-time surface temperature and thermal images of the samples. Mass changes were monitored using an analytical balance (JJ224BC, China). Ambient temperature and relative humidity were simultaneously recorded by a hygrothermograph (Cos-03, China). The 3D macrostructure analyses were performed in a Zeiss Xradia 610 Versa 3D microCT with a voltage of 80 kV and a power of 10 W. The obtained images were reconstructed by Carl Zeiss’s Object Research Systems (ORS) image analysis program. The microstructure of CB powders was examined by transmission electron microscopy (TEM, Hitachi HT7800) operated at an accelerating voltage of 120 kV. X-ray photoelectron spectroscopy (XPS) was carried out using an ESCALAB 250Xi spectrometer equipped with a monochromatic Al Kα X-ray source. Porosity and pore-size distribution were characterized by mercury intrusion porosimetry (MIP; Autopore IV 9500, Micromeritics). Compressive mechanical tests were performed on a universal testing machine (Z1.0, Zwick) with a 1.0 kN load cell. Raman spectra were collected on a Renishaw inVia Reflex spectrometer using a 532 nm laser; each spectrum was acquired with an integration time of 10 s at 1.2 mW (single accumulation).

## 3 Results and Discussion

Ideally, a solar-powered evaporator should have efficient thermal management with robust water transport to ensure continuous photothermal conversion and stable evaporation. As illustrated in Figure 1a, the designed PDMAPS-CB-SH achieves efficient evaporation in seawater through the antipolyelectrolyte effect of the zwitterionic polymer network. When salt ions penetrate the hydrogel, the porous framework expands due to ion–polymer interactions, leading to moderate volume swelling. This enlarged pore structure facilitates rapid and stable water transport, thereby supporting stable evaporation under the tested saline conditions (e.g., 10 wt % brine). Moreover, the abundant hydrogen bonding sites in the hydrogel matrix reduce the enthalpy of water evaporation by modulating water–polymer interactions, further enhancing energy conversion efficiency. Ice was selected as an environmentally friendly and easily removable porogen to engineer the macroporous architecture of the hydrogels (Figure 1b).^41,42^ During ice-templated polymerization, monomers are excluded into confined regions between growing ice crystals and subsequently polymerize in situ, yielding a sponge-like hydrogel with interconnected pores after ice removal. Since the size of ice crystals is temperature-dependent, the pore dimensions of the hydrogel can be tuned by controlling the prefreezing temperature. This strategy enables the fabrication of polyzwitterionic hydrogels with optimized pore structures for efficient and stable solar evaporation in saline environments.

**Figure 1:**
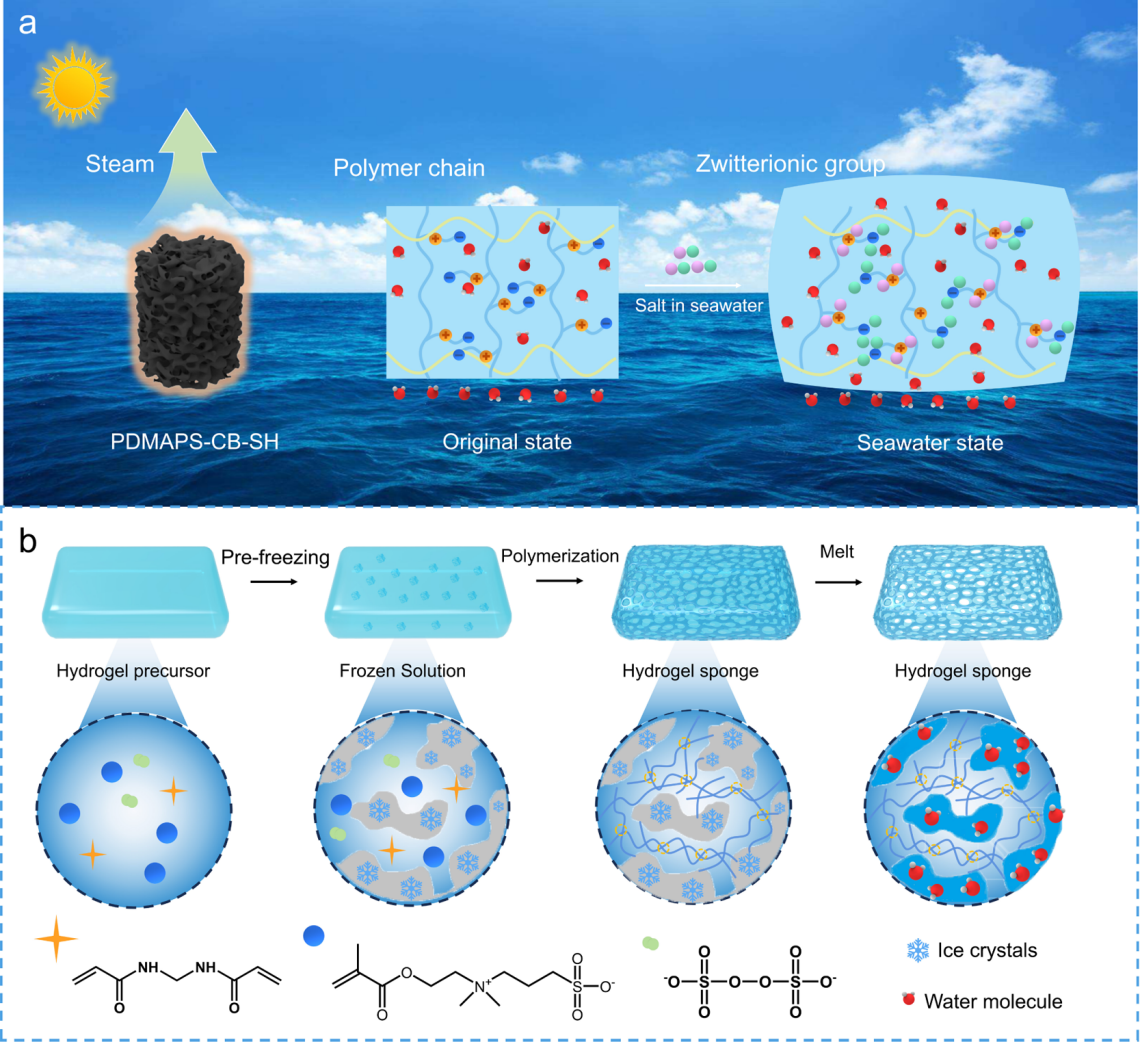
(a) Schematic illustration of the PDMAPS-CB-SH evaporator with a porous sponge-like structure for integrated solar-driven desalination in brines. Owing to the antipolyelectrolyte effect, the polyzwitterionic hydrogel can absorb and swell in high-salinity brines, enabling stable evaporation performance. (b) Schematic representation of the synthesis strategy of PDMAPS-CB-SH.

The water transport performance of photothermal hydrogels is intrinsically governed by their internal pore architecture. By employing ice-templated polymerization, we achieved systematic tuning of pore morphology for PDMAPS-based hydrogels through systematic regulation of the prefreezing temperature. Mercury intrusion porosimetry (MIP) corroborated the well-developed porous architecture of the hydrogel prepared at −30 °C, showing a high total porosity of 40.6% and an average pore diameter of 12.1 μm, consistent with the macroporous features observed by microscopy. Notably, the pore network also exhibits high connectivity, reflected by a relatively low tortuosity (3.2) and a high permeability (40,151 mD), which together provide a structural basis for efficient mass transport through the hydrogel. As shown in Figure 2a, the hydrogel prepared under conventional room-temperature polymerization displayed a crater-like morphology with limited porosity and no obvious interconnected channels. In contrast, hydrogels synthesized under controlled freezing exhibited well-defined, interconnected porous structures. At 0 °C, small pore networks (2–5 μm) began to emerge (Figure 2b), which progressively enlarged as the prefreezing temperature decreased to −10, −20, and −30 °C (Figure 2c–e). The maximum pore size (10–15 μm) was obtained at −30 °C, beyond which further lowering to −40 °C did not induce additional pore enlargement (Figure 2f).

**Figure 2:**
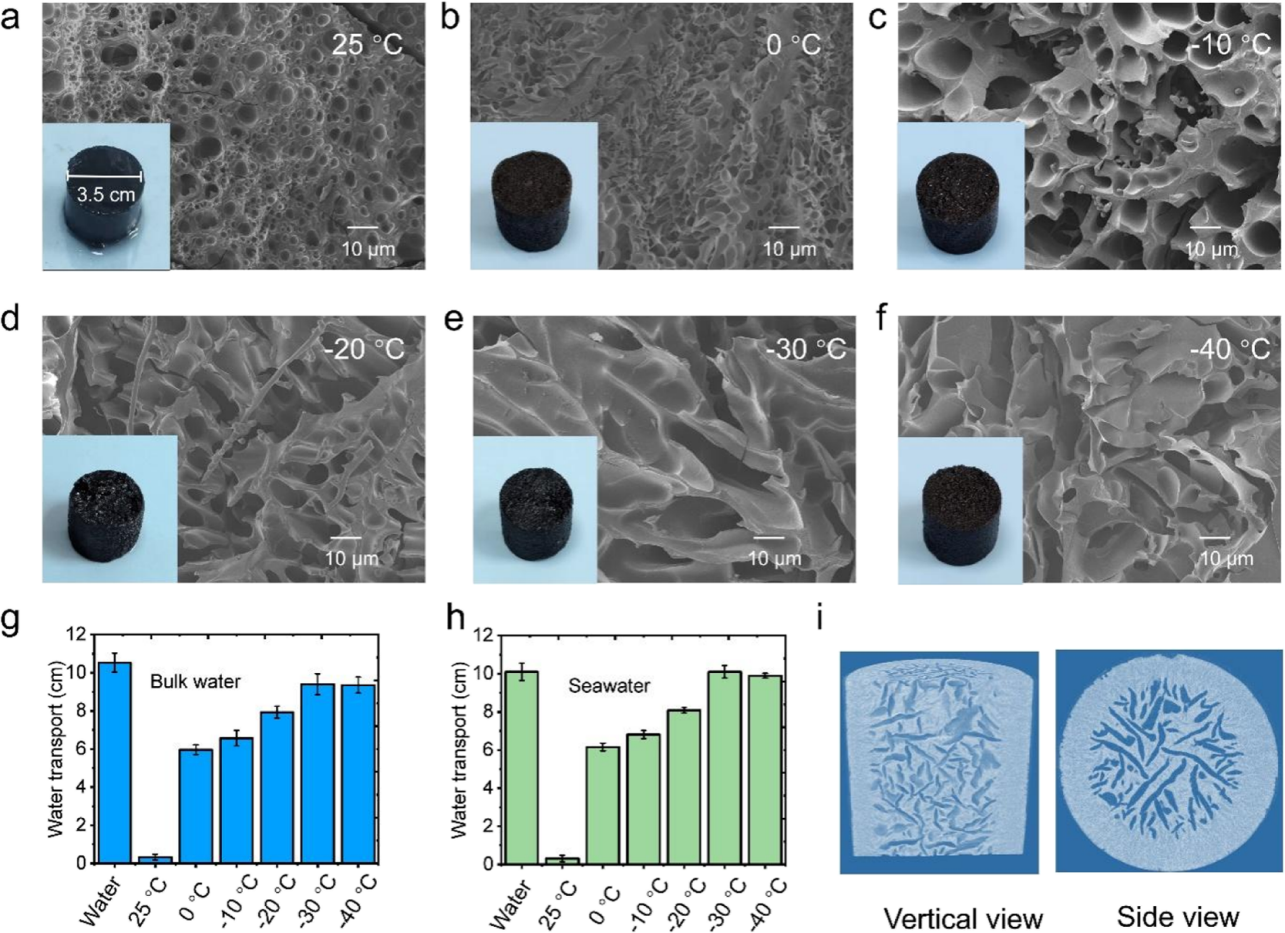
Structural and water transport characterization of PDMAPS-CB hydrogels prepared at different prefreezing temperatures. (a) Photograph and SEM image of hydrogel prepared at 25 °C showing irregular, noninterconnected morphology. (b–f) Photographs and SEM images of hydrogels synthesized at 0, −10, −20, −30, and −40 °C, respectively, revealing progressively enlarged and interconnected pores. (g) Water transport performance in bulk water and (h) seawater, showing maximum efficiency at −30 °C. (i) 3D CT reconstruction of the hydrogel prepared at −30 °C, confirming the formation of a well-defined interconnected pore network.

To correlate these morphological features with transport behavior, a strip-assisted capillary uptake experiment was conducted (Figure S1). The hydrogel fabricated at 25 °C exhibited the poorest water transport, reaching only ∼3.2% of the bulk water reference. In stark contrast, the spongy hydrogel obtained at −30 °C achieved a high transport efficiency (∼89.3% of bulk water, Figure 2g). The same trend persisted in seawater (3.5 wt %), where the hydrogel prepared at −30 °C maintained superior transport capacity over all other samples (Figure 2h). These results can be rationalized by the ice crystals growth mechanism: the morphology of ice crystals directly dictates the resulting pore architecture. At higher freezing temperatures (above −10 °C), the slow cooling rate and low degree of supercooling limit nucleation events, leading to uneven, closed-pore structures that hinder water transport. As the freezing temperature decreases, more nucleation sites form, generating increasingly uniform and interconnected channels. Once the temperature reaches −30 °C, the pore structure and corresponding water transport performance stabilize. 3D micro-CT reconstruction further verifies the highly interconnected porous network of the optimized hydrogel (Figure 2i), confirming that pore size engineering plays an important role in enabling efficient water delivery and sustaining stable evaporation under both freshwater and saline conditions. The mechanical robustness of the hydrogels was further assessed by rheological measurements (Figure S2). Both PDMAPS-CB-HG and PDMAPS-CB-SH exhibited comparable storage moduli (*G*′ ∼ 10,000 Pa) over the entire frequency range, indicating similar elastic responses. However, their loss moduli (*G*″) differed significantly: PDMAPS-CB-HG showed relatively high energy dissipation (4600–7000 Pa), whereas PDMAPS-CB-SH displayed much lower values (500–1000 Pa). The reduced viscous component in PDMAPS-CB-SH is attributed to its highly porous structure, which effectively suppresses energy dissipation and contributes to improved mechanical stability.

As shown in Figures S3 and 3a, a solar simulator coupled with an electronic balance was employed to evaluate the photothermal heating behavior and evaporation performance of the samples in both bulk water and brine. The PDMAPS-CB-SH was mounted on polystyrene (PS) foam to ensure stable floating during the tests. The FTIR spectrum of PDMAPS-CB-SH (Figure S4) displayed distinct absorption bands corresponding to O–H (3400 cm^–1^), C=O (1722 cm^–1^), N–H (1481 cm^–1^), and −SO_3_^–^ (1168 and 1038 cm^–1^), supporting the successful incorporation of the zwitterionic monomers into the hydrogel network. XPS was further performed on the pristine hydrogel (PDMAPS-SH) and the carbon-black-modified hydrogel (PDMAPS-CB-SH) to examine the surface chemical composition (Figure 3b). High-resolution C 1s spectra and peak deconvolution reveal components assignable to C–C (284.80 eV), C–N (∼286.0 eV), C–O (∼287.1 eV), and C=O (∼288.7 eV), consistent with the polymer framework. After CB modification, the sp^2^ carbon contribution at ∼284.0 eV (sp^2^ C=C) becomes more prominent, indicating the successful introduction of CB on the hydrogel surface. Consistently, optical measurements (Figures 3c and S5) show that incorporating CB markedly enhances solar absorption across the visible and near-infrared regions while reducing reflection. The raw CB powder was further characterized by TEM-EDS (Figure S6), confirming nanoscale primary particles with typical aggregation. Notably, the porous sponge-like structure of PDMAPS-CB-SH promotes multiple light scattering, further improving light harvesting. Infrared thermography verifies its superior photothermal response: under 1 sun irradiation, the surface temperature of PDMAPS-CB-SH is 13.8 °C higher than that of the undoped PDMAPS-SH (Figure 3d,e).

**Figure 3:**
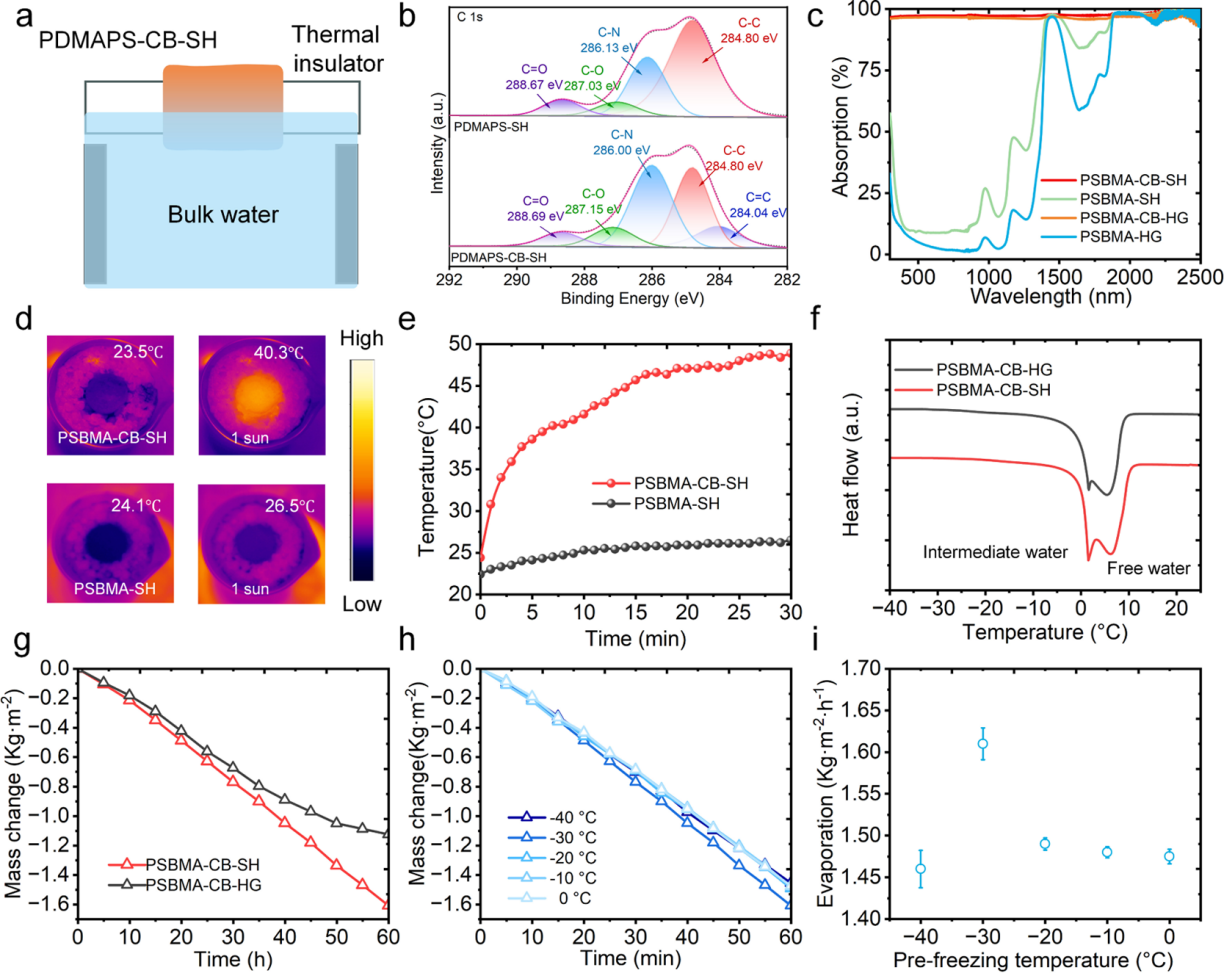
Photothermal and solar-driven evaporation performance of PDMAPS-based hydrogels under 1 sun (1.0 kW m^–2^). (a) Schematic of the laboratory solar evaporation setup and hydrogel floating configuration. (b) High-resolution XPS C 1s spectra of PDMAPS-SH and PDMAPS-CB-SH. (c) UV–vis-NIR absorption spectra of PDMAPS-HG, PDMAPS-CB-HG, PDMAPS-SH, and PDMAPS-CB-SH. (d) Infrared thermal images and (e) corresponding surface temperature profiles of hydrogels with and without CB. (f) DSC curves revealing water states (intermediate and free water) in PDMAPS-CB-HG and PDMAPS-CB-SH. (g) Mass change curves of PDMAPS-CB-HG and PDMAPS-CB-SH under solar irradiation. (h–i) Evaporation performance of PDMAPS-CB-SH prepared at different prefreezing temperatures.

To assess the leaching risk of CB during prolonged water contact, a long-term immersion test was conducted by soaking the composite hydrogel in deionized water for 10 days. The supernatant remained visually clear with no observable turbidity or color change (Figure S7). UV–vis spectroscopy provides further evidence: the 10-day leachate shows a nearly flat, low absorbance profile over 400–800 nm and closely overlaps with pure water, whereas CB dispersions (0.01–1 ppm) exhibit substantially higher absorbance over the same range (Figure S8). These results indicate negligible CB leaching and suggest that CB is stably retained within the hydrogel network under aqueous conditions relevant to practical operation. To elucidate the evaporation mechanism, the water states within the hydrogels were analyzed by DSC (Figure 3f). Three distinct states (i.e., bound water, intermediate water, and free water) govern the evaporation enthalpy. Notably, PDMAPS-CB-SH contained a higher fraction of intermediate water compared with PDMAPS-CB-HG, resulting in a reduced phase-change enthalpy.^43^ This reduction lowers the energy barrier for evaporation, directly contributing to higher evaporation efficiency. To assess the hydration microenvironment, Raman spectra were recorded for the fully hydrated PDMAPS-CB-SH hydrogel and the O–H stretching band was deconvoluted into three components. Relative contents were calculated by peak-area integration of the fitted peaks, yielding 8.63% bound water, 51.12% intermediate water, and 40.25% free water (Figure S9). Continuous solar irradiation experiments corroborated this mechanism: although PDMAPS-CB-HG absorbed slightly less light, it exhibited uncontrolled surface heating, ultimately reaching ∼49 °C due to insufficient water replenishment at the evaporating interface. In contrast, the spongy PDMAPS-CB-SH maintained effective water transport, preventing surface dehydration and stabilizing the interface temperature at ∼40 °C (Figure S10a). Consequently, PDMAPS-CB-SH achieved an evaporation rate 1.52 times that of PDMAPS-CB-HG under identical conditions (Figure 3g). Finally, the influence of prefreezing temperature on performance was systematically investigated. While all hydrogels exhibited comparable photothermal conversion efficiency in the hydrated state (Figure S10b), the evaporation rates varied significantly with pore architecture. As shown in Figure 3h–i, the hydrogel prepared at −30 °C displayed the optimal evaporation performance, in excellent agreement with the water transport characteristics described above. These results highlight the synergistic role of reduced enthalpy change and efficient water supply in enabling stable and high-rate solar evaporation.

After identifying the optimal hydrogel configuration, we systematically investigated the salt tolerance and evaporation behavior of PDMAPS-CB-SH in brine environments.^44^ As illustrated in Figure 4a,b, salt ions permeating the zwitterionic network screen the strong intra/inter-chain electrostatic (dipolar) associations between oppositely charged moieties, thereby relaxing the polymer chains. This relaxation induces volumetric expansion and enlarges internal water/ion transport pathways, which facilitates continuous water replenishment to the evaporation interface and is beneficial for evaporation in saline media. Quantitative swelling results (Figure 4c) verify this antipolyelectrolyte behavior: the volumetric expansion increases monotonically with NaCl concentration and reaches ∼192% at 20–25 wt %. Importantly, this salt-induced swelling mechanism is distinct from the Donnan-osmotic swelling typically invoked for conventional polyelectrolytes. As summarized in Figure S11, ICP-OES was used to quantify ion uptake/association and to estimate the salt concentration inside the hydrogel at swelling equilibrium. When the dry gel was immersed in 10 wt % NaCl and allowed to equilibrate, the internal salt concentration reached 8.33 wt %, close to the external concentration, indicating that ions can freely enter and leave the network. Therefore, the Donnan osmotic pressure contribution is negligible, and the observed “salt-induced swelling” primarily originates from the screening of dipole–dipole interactions at high ionic strength, i.e., the classic antipolyelectrolyte effect.

**Figure 4:**
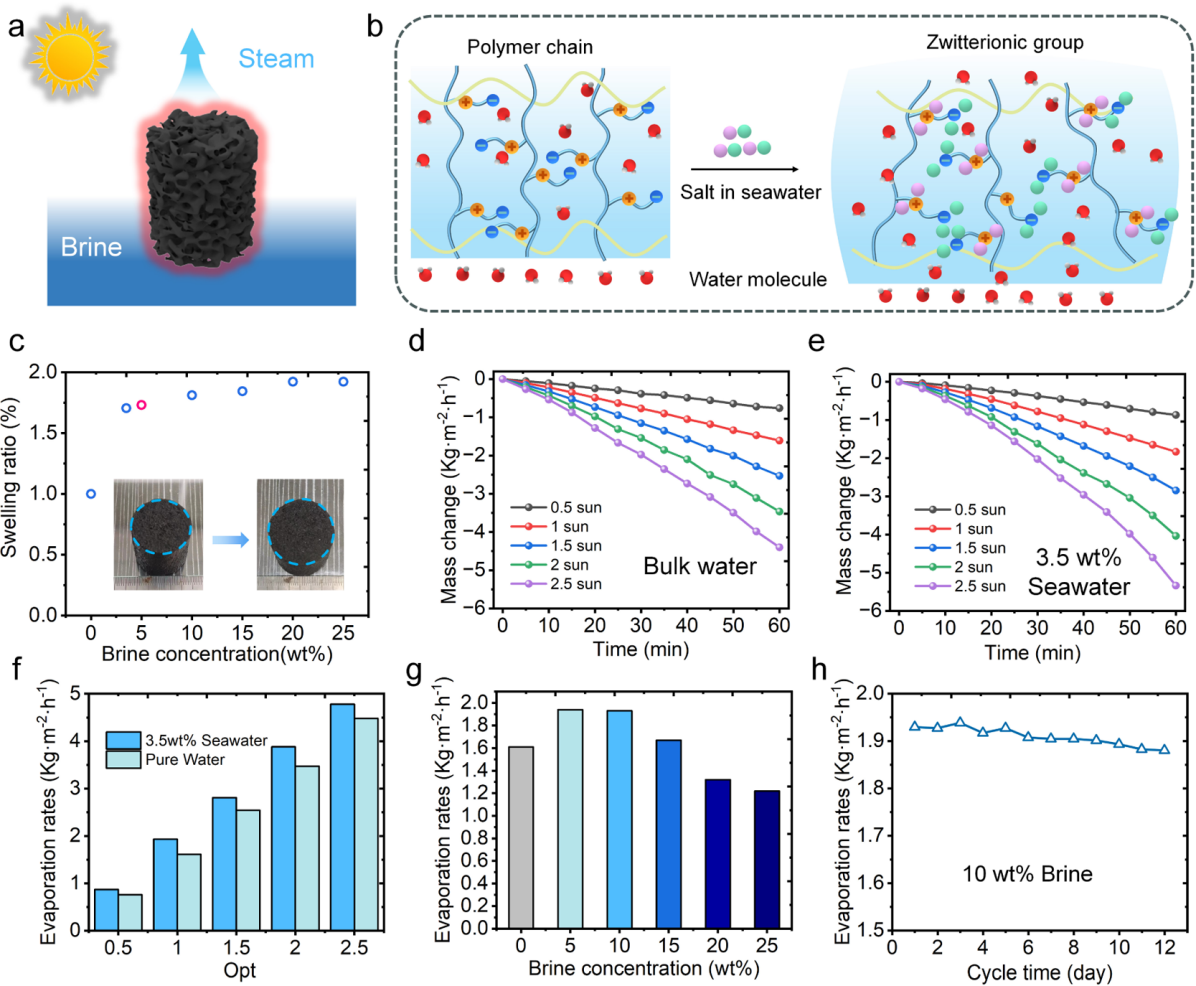
Salt-tolerant solar evaporation performance of PDMAPS-CB-SH. (a,b) Schematic of brine-induced swelling and water transport in the zwitterionic hydrogel. (c) Swelling ratios of PDMAPS-CB-SH at different NaCl concentrations. (d,e) Mass change curves under various solar intensities in bulk water and seawater (3.5 wt %). (f) Evaporation performance comparison between pure water and seawater. (g) Evaporation rates at different brine concentrations. (h) Evaporation rates in 10 wt % brine measured over a 12-day test (8 h solar irradiation and 16 h darkness per day).

We next evaluated evaporation under different solar intensities in both bulk water and seawater (3.5 wt %). As shown in Figure 4d–f, PDMAPS-CB-SH consistently delivers higher evaporation rates in seawater than in pure water across 0.5–2.5 sun. At 1 sun, the evaporation rate reaches 1.93 kg m^–2^ h^–1^ in seawater versus 1.61 kg m^–2^ h^–1^ in bulk water. This enhancement can be attributed to two synergistic factors: (i) a reduced effective evaporation enthalpy in saline media (Table S1),^45^ and (ii) brine-induced swelling that enlarges transport channels and accelerates water replenishment at the evaporation front.^46^ Consistently, in 10 wt % brine the evaporation rates increase with solar intensity (0.92, 1.93, 3.00, 4.26, and 5.63 kg m^–2^ h^–1^ from 0.5 to 2.5 sun; Figure S12), outperforming bulk water by ∼20% across the tested conditions. Benchmarking against reported zwitterionic hydrogel-based evaporators,^31,38,47–52^ further indicates that our system achieves comparable performance even under the more stringent 10 wt % brine condition (Figure S13), whose salinity is substantially higher than seawater. At extreme salinities (≥15 wt %), however, the evaporation rate decreases sharply (e.g., 1.22 kg m^–2^ h^–1^ at 25 wt %; Figure 4g), mainly due to surface salt crystallization that blocks transport pathways and restricts water supply (Figure S14).

We next quantified the evaporation efficiencies of PDMAPS-CB-SH under various light intensities. As shown in Figure S15, efficiencies in seawater (86.3–95.1%) closely matched those in bulk water (88.3–95.3%), demonstrating that the zwitterionic hydrogel maintains high conversion efficiency even under saline conditions. Notably, the slightly lower values in brine are consistent with its reduced enthalpy of evaporation (Table S1). Long-term cycling tests further confirmed the operational stability of PDMAPS-CB-SH in concentrated brines. Under intermittent irradiation (8 h light/16 h dark), the hydrogel maintained a relatively stable evaporation rate in 10 wt % saline, with an average rate of 1.88 kg m^–2^ h^–1^ over 12 days (Figure 4h). When further challenged in 20 wt % saline, more pronounced salt crystallization occurs after 8 h irradiation, causing a temporary performance drop (Figure S16); notably, the surface salt readily dissolves during the subsequent dark period, enabling autonomous recovery of evaporation capacity (Figure S17). These results underscore the self-regulating desalination behavior of PDMAPS-CB-SH under high salinity. Finally, compressive tests before and after repeated desalination/evaporation cycles show that the mechanical response remains largely unchanged after 3, 6, 9, and 12 cycles (Figure S18a,b), with only a slight stress decrease at the same strain. The cycled samples still sustain ∼30 kPa-level compressive stress at moderate strains, indicating that the porous framework and structural integrity are well preserved during repeated operation.

To assess practical desalination capability, the optimized PDMAPS-CB-SH was tested in real seawater under natural sunlight. Scaled-up evaporators were readily obtained by increasing the mold size, and both PDMAPS-CB-SH and the nonporous control PDMAPS-CB-HG were integrated into a portable SSG device (Figure 5a–c). In this setup, the hydrogel evaporator floated on the seawater surface inside a transparent condensation hood; the generated vapor condensed on the inner cover and was collected via gravity-driven channels into a graduated vessel. Outdoor tests were conducted in Ningbo on a clear day (June 4, 2025; 9:00–18:00). Solar irradiance, ambient temperature, and relative humidity were monitored hourly using a solar radiometer and thermohygrometer (Figure 5d–f), with average values of 487 W m^–2^, 32.4 °C, and 37.3%, respectively. Under these conditions, PDMAPS-CB-SH produced ∼34.9 g of freshwater over 9 h. Notably, owing to its three-dimensional architecture, the evaporator provides a substantially enlarged effective evaporation area compared with its projected footprint, analogous to natural plants that extend vertically to increase functional surface area. As quantified in Figure S19, the actual evaporation area of PDMAPS-CB-SH (top + side surfaces) is 47.1 cm^2^, whereas the projected area is 28.26 cm^2^. Accordingly, the same outdoor yield corresponds to 7.41 kg m^–2^ day^–1^ when normalized by the actual area and 12.35 kg m^–2^ day^–1^ when normalized by the projected area. This comparison clarifies why the projected-area-normalized value appears higher for the 3D configuration. In contrast, the 2D nonporous PDMAPS-CB-HG generated only 12.9 g of freshwater during the same test, corresponding to 4.56 kg m^–2^ day^–1^ (projected-area normalization), i.e., ∼37% of the output of PDMAPS-CB-SH under identical environmental conditions (Figure 5g). This performance disparity is attributed to the absence of an interconnected porous network in PDMAPS-CB-HG, which restricts capillary replenishment from the bulk and leads to surface dehydration under irradiation. This behavior is directly supported by the visibly dried top surface of HG compared to the persistently moist interface of SH (Figure 5h). Finally, the quality of the collected condensate was evaluated by ICP-OES. Relative to the feed seawater, the concentrations of Na^+^, K^+^, Ca^2+^, Mg^2+^, Sr^2+^, and B (reported as elemental B) were reduced by more than 3 orders of magnitude, meeting WHO drinking-water guidelines (Figure 5i). Collectively, these results demonstrate scalable fabrication and outdoor desalination performance under the tested field conditions.

**Figure 5:**
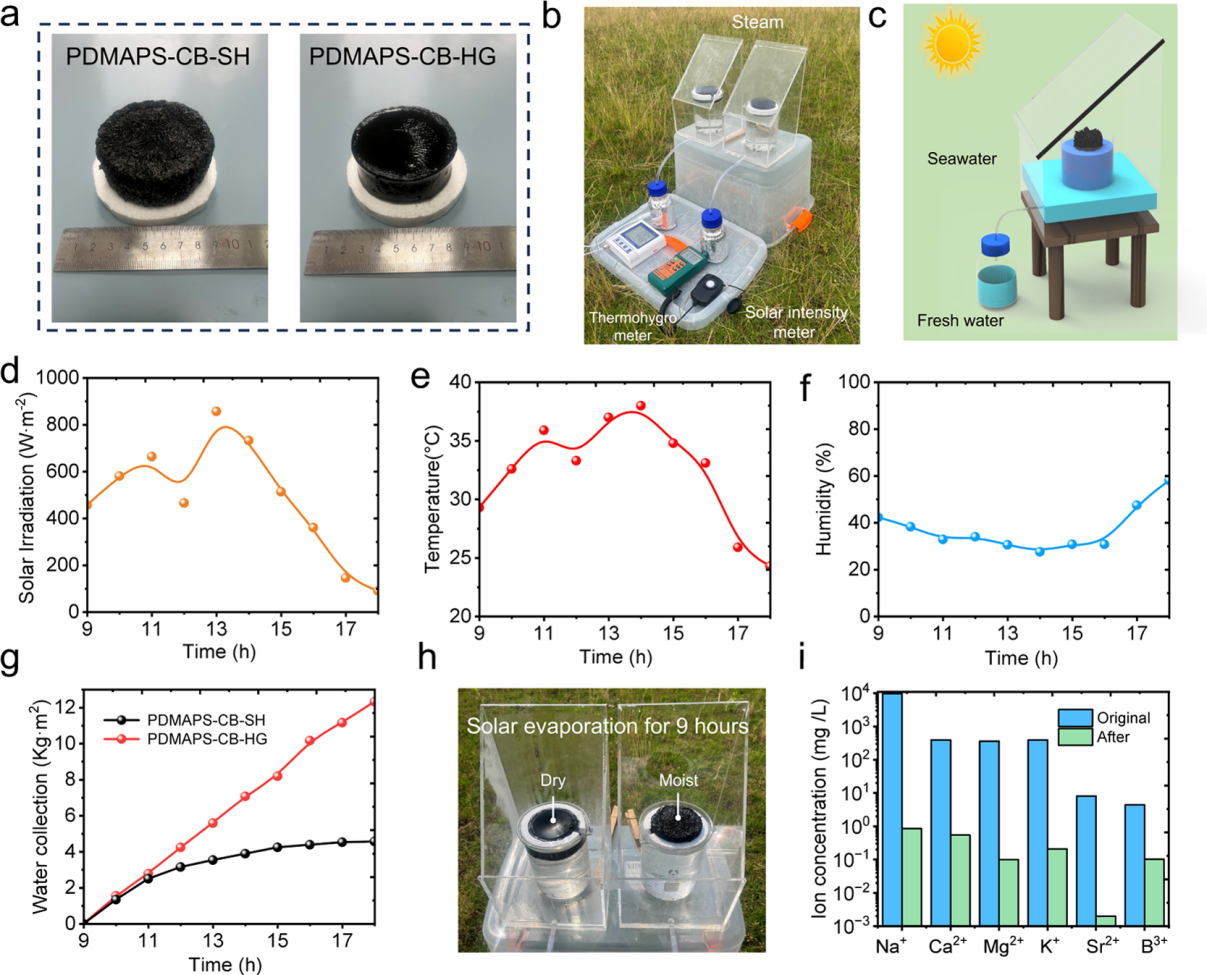
Outdoor desalination performance of PDMAPS-CB-SH under natural sunlight. (a) Scaled-up PDMAPS-CB-SH and PDMAPS-CB-HG evaporators (ca. 6 cm diameter). (b) Portable outdoor SSG (solar steam generation) device with onboard thermohygrometer and solar radiometer. (c) Schematic of the floating evaporator and condensation and collection pathway. (d–f) Hourly solar irradiance, ambient temperature, and relative humidity during the field test (9:00–18:00). (g) Cumulative freshwater yield, normalized to absorber area for PDMAPS-CB-SH and PDMAPS-CB-HG. (h) Photographs of the PDMAPS-CB-SH (moist surface) and PDMAPS-CB-HG (dehydrated surface) after 9 h solar evaporation. (i) ICP-OES analysis of major ions (i.e., Na^+^, K^+^, Ca^2+^, Mg^2+^, Sr^2+^, and B) before and after desalination; condensate ion levels are reduced by >10^3^× and meet WHO guidelines.

## 4 Conclusion

In summary, we developed a pore-size-tunable, zwitterionic sponge hydrogel (PDMAPS-CB-SH) via ice-templated polymerization that integrates efficient light harvesting, accelerated water transport, and salt-tolerant evaporation. The interconnected porous framework, optimized at −30 °C prefreezing, enabled rapid capillary pumping and stable surface hydration, while zwitterionic functionalities endowed the hydrogel with antipolyelectrolyte swelling in high-salinity brines. Combined with abundant intermediate water that reduced evaporation enthalpy, the PDMAPS-CB-SH evaporators exhibited markedly enhanced solar steam generation compared with conventional hydrogels. Beyond laboratory validation, outdoor field tests under natural sunlight demonstrated a daily freshwater yield of 12.35 kg m^–2^, three times higher than nonporous controls, with collected water quality meeting WHO drinking standards. The synergy of structural design, molecular functionality, and scalable fabrication highlights PDMAPS-CB-SH as a scalable zwitterionic hydrogel evaporator for solar desalination. This work not only provides mechanistic insights into the role of zwitterionic swelling and intermediate water in solar-driven evaporation but also offers a basis for solar desalination under saline and moderately hypersaline conditions.

## Supplementary Material



## Notes

Including photographs of capillary-driven water transport and rheological properties of PDMAPS-CB-HG and PDMAPS-CB-SH; FT-IR spectrum and surface-temperature evolution of PDMAPS-CB-SH; reflectance and transmittance spectra; photographs of PDMAPS-CB-SH after evaporation in brines with varying concentrations; and solar-evaporation efficiencies of PDMAPS-CB-SH in deionized water and 3.5 wt % seawater; a schematic of the experimental setup for solar evaporation; TEM and EDS analyses of carbon black (CB); long-term stability images of PDMAPS-CB-SH in water; UV–vis absorption data of CB dispersions and leachate; Raman spectra of the hydrogel under saline conditions; ICP-OES results on ion uptake; evaporation performance under different solar intensities and brine concentrations; cycling stability tests; compressive mechanical properties before and after repeated use; a comparison between actual and projected evaporation areas (PDF)
