# Supplementary material for: Ice-Templated Zwitterionic Sponge Hydrogels for Stable and Efficient Solar Desalination in High-Salinity Brines
Source: ACS Appl Mater Interfaces. 2026 Feb 2;18(5):8981–90. doi: 10.1021/acsami.6c00207 (PMC12903110; doi:10.1021/acsami.6c00207)
Supplement: Supplementary file 1 [file am6c00207_si_001.pdf]

Supplementary materials

Ice-Templated Zwitterionic Sponge Hydrogels for Stable and Efficient Solar Desalination in High-Salinity Brines

Chang Zhang[1*], Tiantian Yao[2], Jincui Gu[3], Peng Xiao[3*], Tao Chen[3], Xuanzhou Chen[4], Louis D. Zhang[5, 6*], and Yanhui Zhang[1*]

[1] School of Biological and Chemical Engineering, NingboTech University, Ningbo 315100, China.

[2] Fuan Pharmaceutical Group Ningbo Team Pharmaceutical Co., Ltd., Ningbo 315100, China.

[3] Key Laboratory of Marine Materials and Related Technologies, Zhejiang Key Laboratory of Marine Materials and Protective Technologies, Ningbo Institute of Materials Technology and Engineering, Chinese Academy of Sciences, Ningbo 315201, China.

[4] School of Electrical and Computer Engineering, Georgia Institute of Technology, Atlanta, GA 30332, USA.

[5] College of Engineering, University of Akron, Akron, OH 44325, USA.

[6] Hangzhou Zheren Tech Co., Ltd., Hangzhou 311100, China.

Corresponding authors: C. Zhang (zhangchang@nbt.edu.cn), Y. Zhang (zyh@nbt.edu.cn), L. D. Zhang (dz39@uakron.edu and zhanglouis94@outlook.com), and P. Xiao (xiaopeng@nimte.ac.cn)

Calculation of the enthalpy change of evaporation

$$U_{in} = E_{equ}m_g = E_0m_0 \quad \text{(S1)}$$

where m_g is the mass change of PDMAPS-CB HG or PDMAPS-CB SH under dark conditions, E_0 (2,257 J g^{-1}) is the evaporation enthalpy, and m_0 represents the corresponding mass of evaporated bulk water. The equivalent enthalpy of evaporation (E_{equ}) for different samples and environments was calculated according to Equation (1). The measured values of evaporation enthalpy for the various hydrogel samples in both bulk water and saline environments are summarized in Table S1.

Table S1. Calculated evaporation enthalpy of different hydrogel samples in bulk water and saline water environments.

**Temperature (°C)**	**Materials**	**Condition**	**Enthalpy (J g^{-1})**
25	-	Bulk water	2257
25	-	Sea water	2300
25	PDMAPS-CB HG	Bulk water	2100
25	PDMAPS-CB HG	Sea water	2068
25	PDMAPS-CB SH	Bulk water	1880
25	PDMAPS-CB SH	Sea water	1634

As shown in Table S1, the evaporation enthalpy of PDMAPS-CB-HG was measured to be 2100 J g^{-1} in bulk water and 2068 J g^{-1} in seawater, respectively. In contrast, PDMAPS-CB-SH exhibited significantly lower values of 1880 J g^{-1} in bulk water and 1634 J g^{-1} in seawater, highlighting its reduced energy requirement for water evaporation.

Calculation of the evaporation efficiency

The solar-driven purification experiments were performed under a solar simulator (HM-Xe500W) equipped with an AM 1.5 G filter. The evaporation efficiency (η) was calculated according to Equation (2):

$$\eta = \frac{mE_{equ}}{qC_{opt}} \quad \text{(S2)}$$

where m is the mass flux of hydrogel evaporator, C_{opt} is the optical concentration on the evaporation surface, and q is the nominal solar intensity (1 kW m^{-2}). E_{equ} represents the equivalent evaporation enthalpy of water in the hydrogel evaporator, which was determined under dark conditions assuming the same energy input (U_{in}).

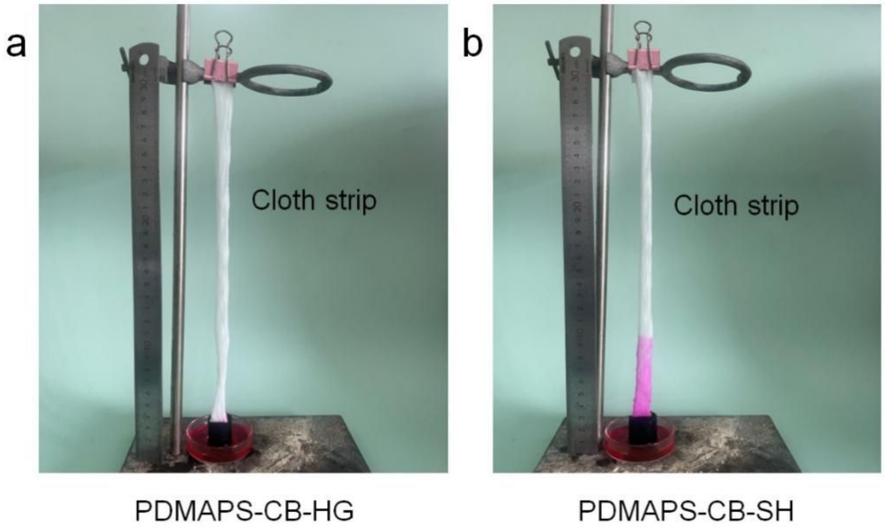

Figure S1. Photographs of the water transport experiments comparing (a) PDMAPS-CB-HG and (b) PDMAPS-CB-SH.

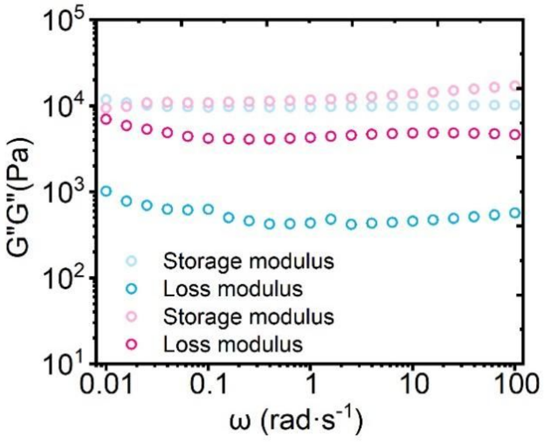

Figure S2. Rheological properties of PDMAPS-CB-HG (red) and PDMAPS-CB-SH (blue), showing storage modulus (G') and loss modulus (G'') as a function of frequency.

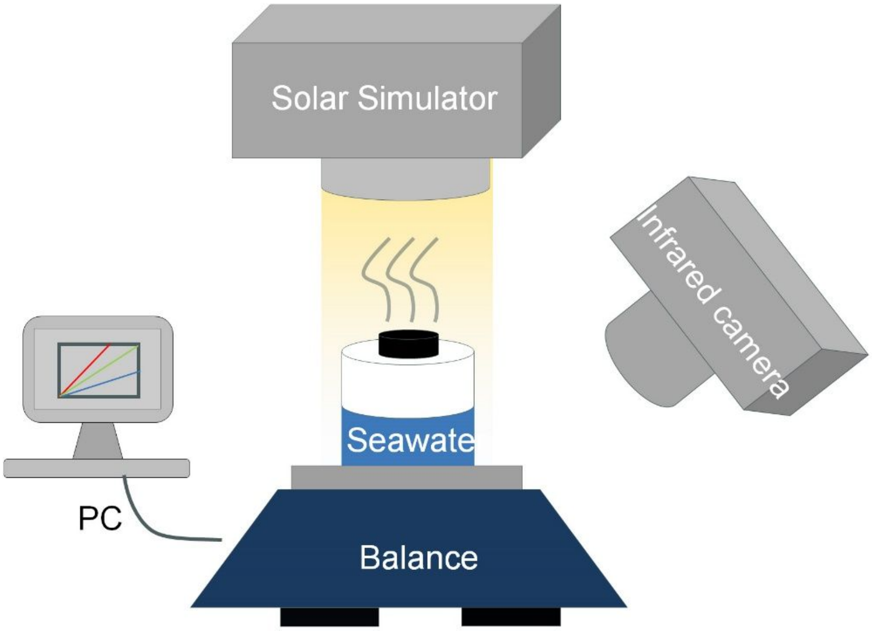

Figure S3. Schematic of the laboratory solar evaporation setup and hydrogel floating configuration.

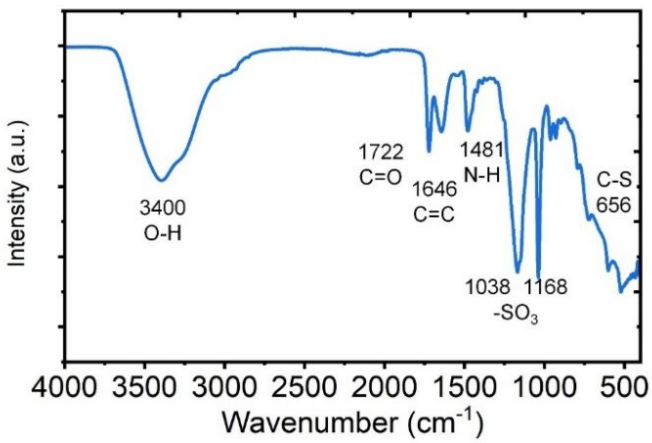

Figure S4. FT-IR spectrum of PDMAPS-CB-SH.

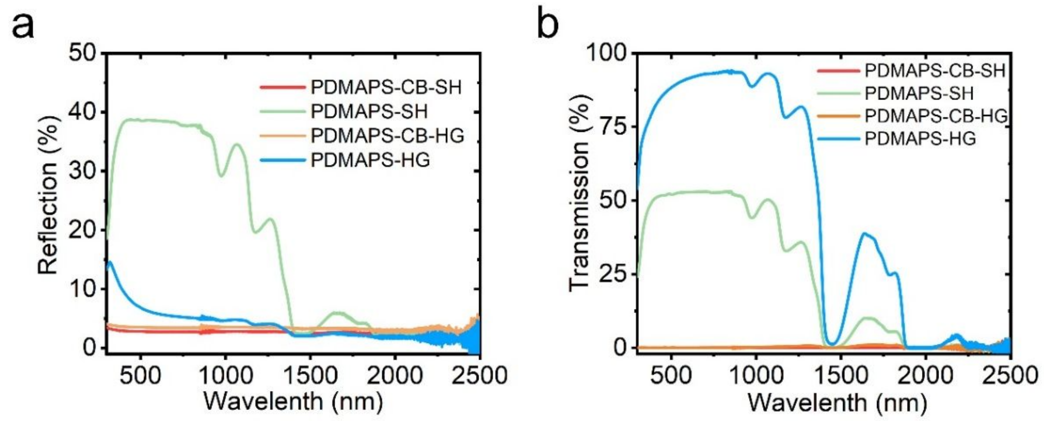

Figure S5. (a) Reflection and (b) transmission spectra of PDMAPS-SH, PDMAPS-SH, PDMAPS-CB-SH, and PDMAPS-CB-SH.

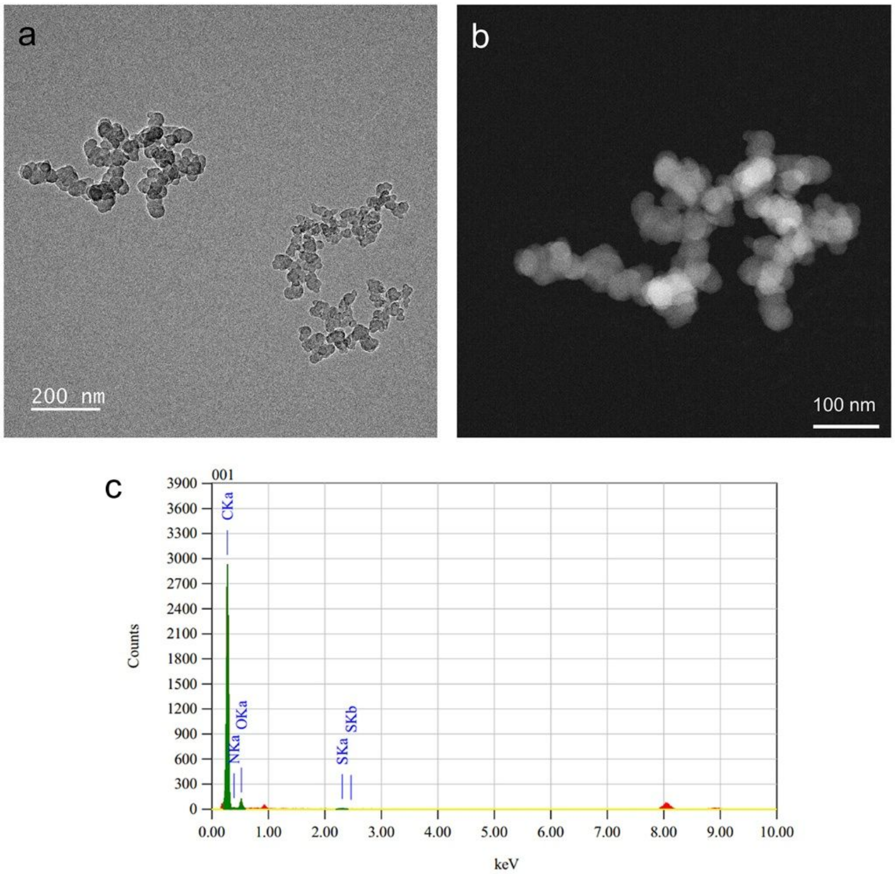

Figure S6. (a) Representative TEM image of CB. (b) Magnified view with the EDS acquisition area marked. (c) Corresponding EDS spectrum of CB showing its elemental composition.

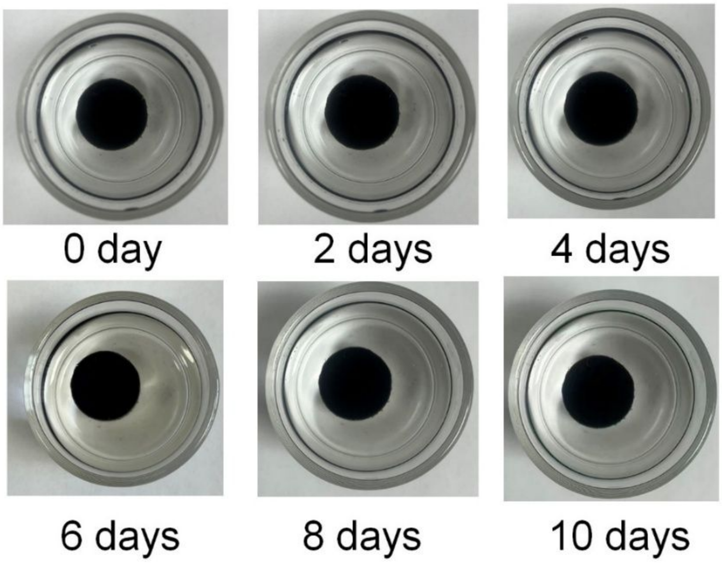

Figure S7. Photographs of the long-term immersion test of the PDMAPS-CB-SH hydrogel in deionized water, recorded at 0, 2, 4, 6, 8, and 10 days.

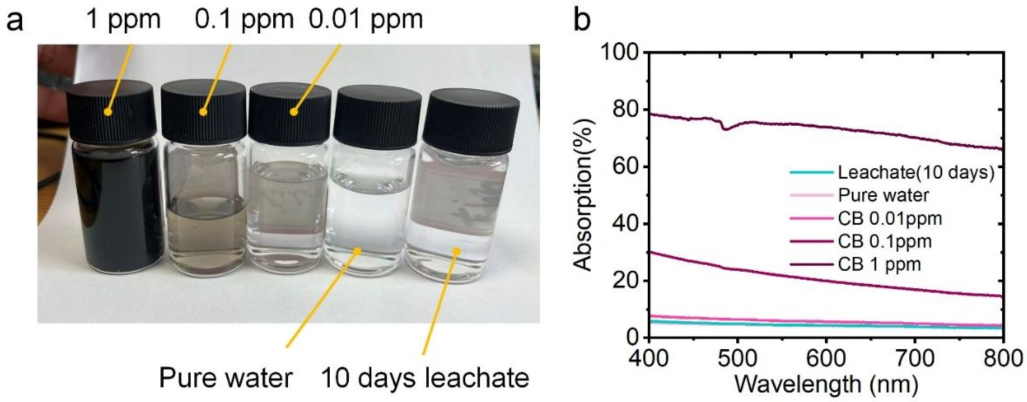

Figure S8. (a) Photographs of CB dispersions (0.01, 0.1, and 1 ppm), pure water, and the leachate collected after 10 days. (b) UV-Vis absorption spectra of CB dispersions at the same concentrations, pure water, and the 10-day leachate.

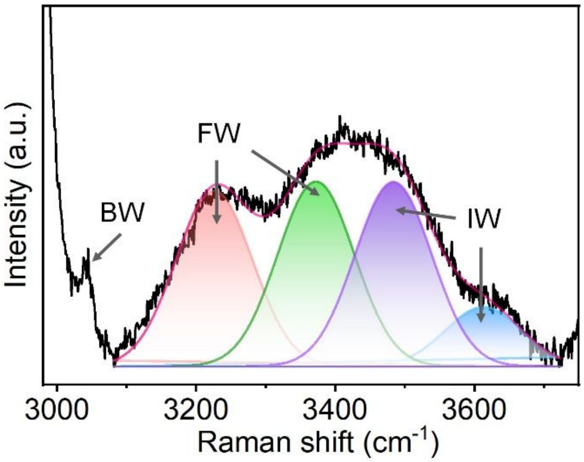

Figure S9. Raman spectra showing the fitting peaks representing bound water (BW), intermediate water (IW), and free water (FW) in the PDMAPS.

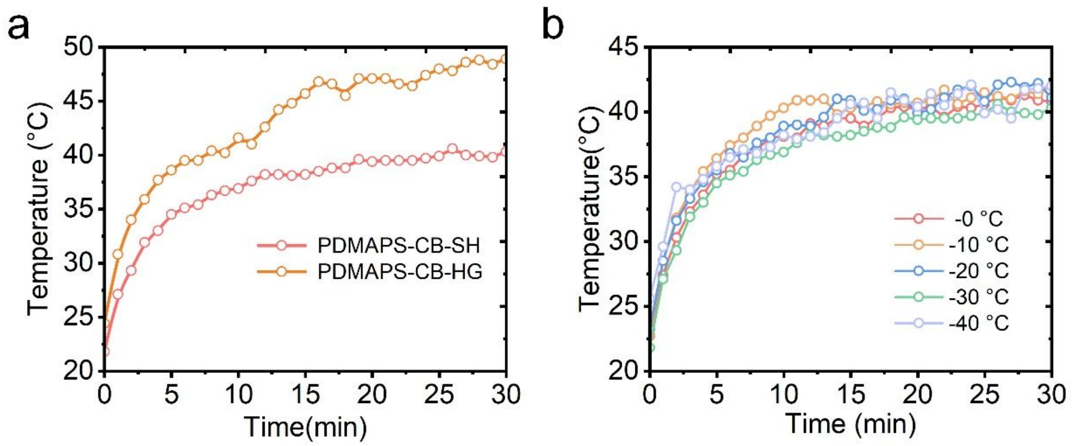

Figure S10. (a) Surface temperature evolution of PDMAPS-CB-HG and PDMAPS-CB-SH under 1 sun irradiation. (b) Surface temperature evolution of PDMAPS-CB-SH prepared at different pre-freezing temperatures under 1 sun irradiation.

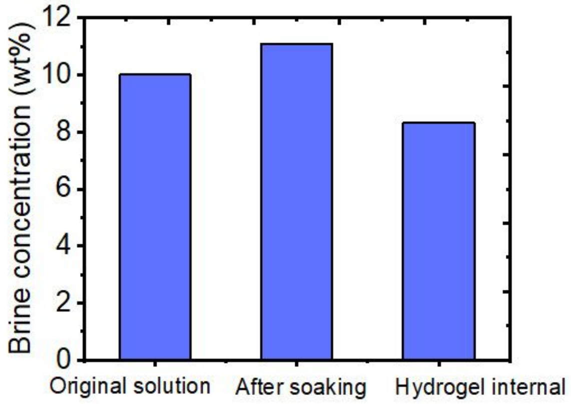

Figure S11. ICP-OES results quantifying the uptake/association of representative ions from 10 wt% brine by the zwitterionic sponge hydrogel. Ion-derived brine concentration (wt%) is compared among the original solution, the supernatant after soaking, and the hydrogel interior, confirming ion uptake/association in the network.

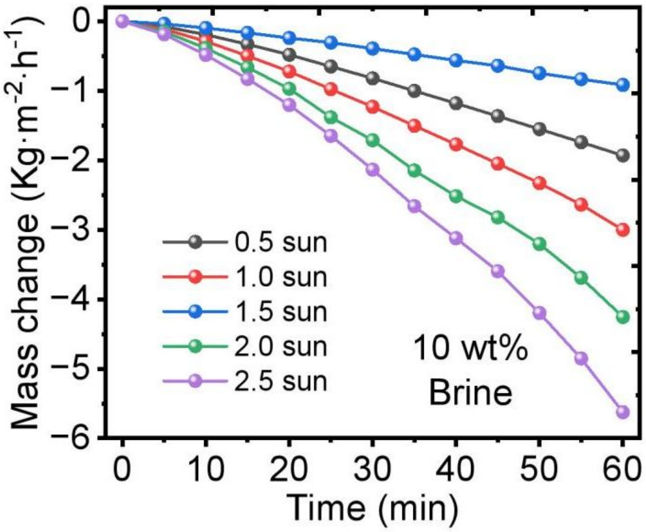

Figure S12. Mass change curves of PDMAPS-CB-SH in 10 wt% brine under different solar intensities (0.5-2.5 sun).

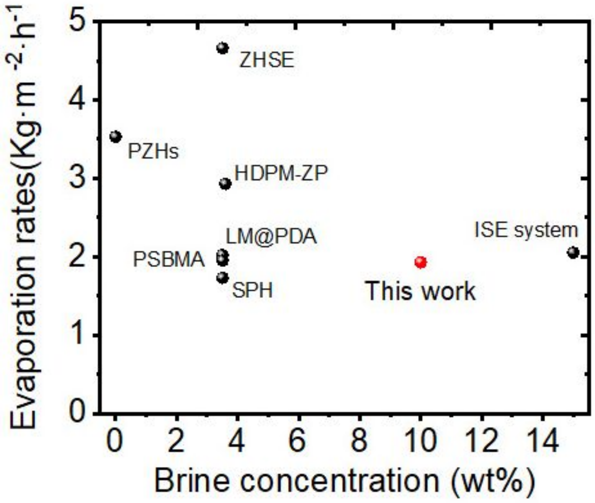

Figure S13. Comparison of evaporation rates for zwitterionic hydrogel evaporators at different brine concentrations.

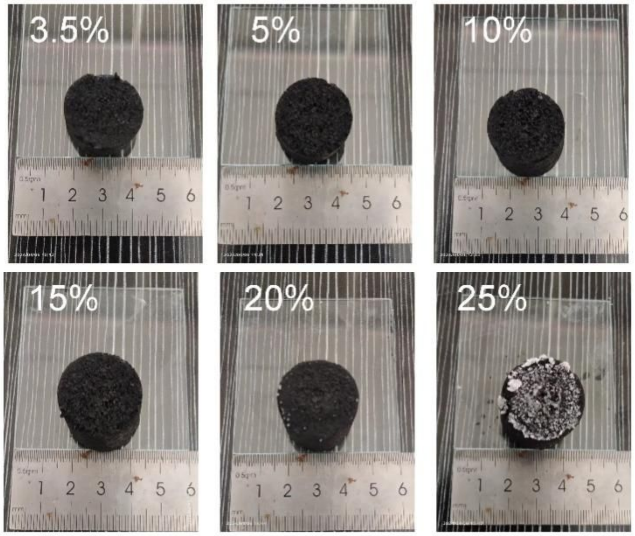

Figure S14. Photographs of PDMAPS-CB-SH after evaporation in brines with different concentrations.

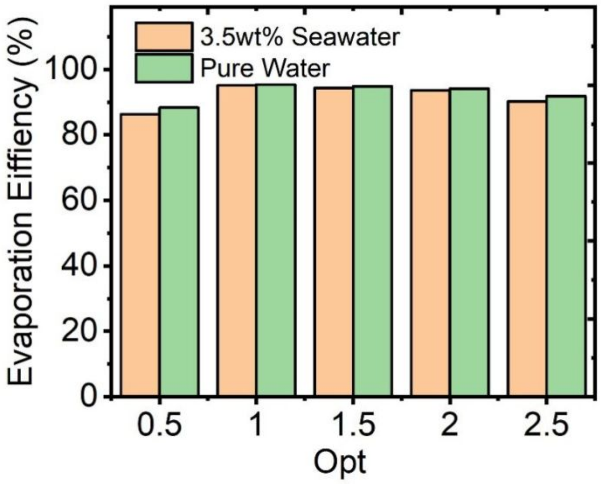

Figure S15. Calculated evaporation efficiencies of DMAPS-CB-SH in pure water and 3.5 wt% seawater under different solar intensities.

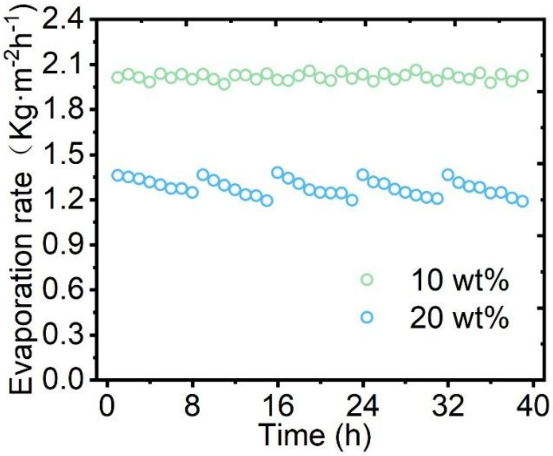

Figure S16. Cycling stability of PDMAPS-CB-SH in 10 wt% and 20 wt% brines under intermittent solar irradiation.

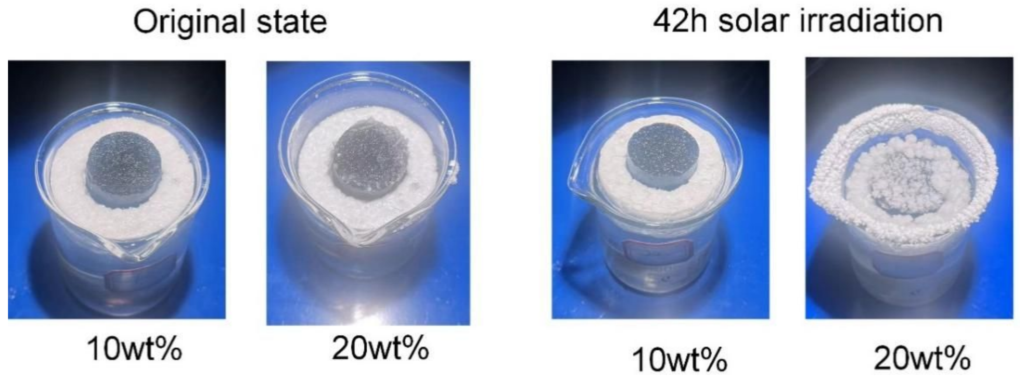

Figure S17. Photographic comparison of PDMAPS-CB-SH before and after 5 evaporation cycles in 10 wt% and 20 wt% brines.

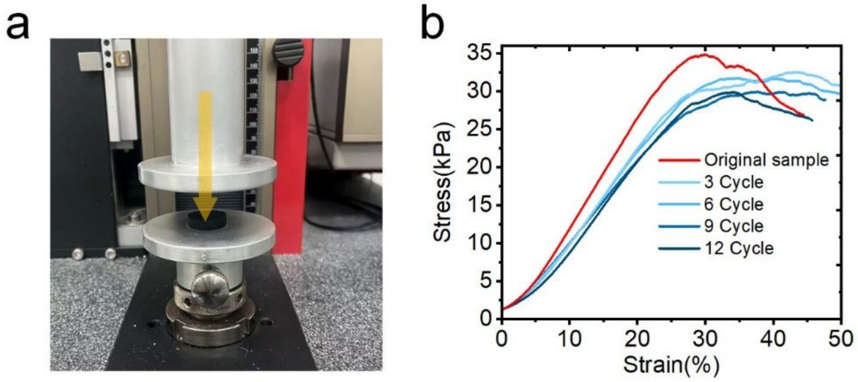

Figure S18. (a) Photograph of the compression test setup; (b) compressive stress-strain curves of the original sample and samples after 3, 6, 9, and 12 operation cycles.

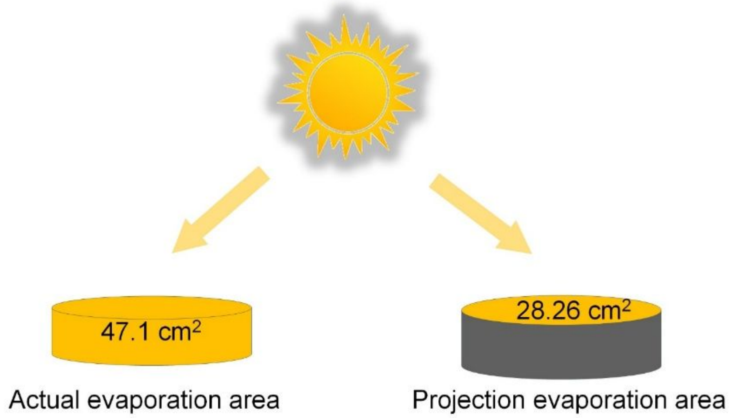

Figure S19. Comparison between actual evaporation area and projected evaporation area.
